# Do antidepressants lead to weight-increase? Antidepressant therapy and long-term changes in body mass index, waist circumference and fat mass - A prospective, population-based study

**DOI:** 10.1192/j.eurpsy.2024.94

**Published:** 2024-08-27

**Authors:** M.-P. Strippoli, M. Preisig

**Affiliations:** Psychiatry, Lausanne University Hospital and University of Lausanne, Prilly, Switzerland

## Abstract

**Abstract:**

The presentation will focus on long-term weight changes in patients with major depressive disorder who use antidepressants. Research studying weight change over periods of more than 12 months is scarce and the effects of depressive episodes and antidepressants on weight changes have rarely been assessed simultaneously. Using data of a prospective population-based CoLaus|PsyCoLaus study, data on the associations of antidepressant use prior to baseline and during a 5.5-year follow-up with changes in adiposity markers and multiple adjustments including for the effects of depressive episodes will be presented. The cohort included 2479 randomly selected 35 to 66 year-old white residents (mean age 49.9 years, 53.3% women) of an urban area who accepted the physical and psychiatric evaluations at baseline and follow-up (76.8% participation at the follow-up). Diagnostic information on mental disorders, treatment use including psychotropic drugs was elicited using a semi-structured interview. Independently of the effect of antidepressants used during the follow-up and the effects of depressive episodes, the number of any antidepressant compounds used prior to baseline was associated with lower increase of body mass index (BMI), whereas the use of antidepressants during the follow-up was associated with steeper increase in BMI and waist circumference. Within AD classes, the use of tricyclic AD (TCA) and selective serotonin reuptake inhibitor (SSRI) prior to baseline was associated with lower increase, the use of SSRI during follow-up was associated with steeper increases in BMI. Similarly, the use of SSRI prior to baseline was associated with lower increase, the use of TCA and SSRI during the follow-up was associated with steeper increase in waist circumference. Finally, the use of SSRI during follow-up was also associated with steeper increase in fat mass. The findings support unfavorable obesogenic effects of sustained treatment not only with TCAs but also with SSRIs, suggesting that the benefit of long-term administration of these AD classes should be carefully weighed against the potential risk of weight gain.

**Disclosure of Interest:**

None Declared

